# The Direct Anterior Approach (DAA) as a Standard Approach for Total Hip Arthroplasty (THA) in Coxa Profunda and Protrusio Acetabuli? A Radiographic Analysis of 188 Cases

**DOI:** 10.3390/jcm12123941

**Published:** 2023-06-09

**Authors:** Tizian Heinz, Hristo Vasilev, Philip Mark Anderson, Ioannis Stratos, Axel Jakuscheit, Konstantin Horas, Boris Michael Holzapfel, Maximilian Rudert, Manuel Weißenberger

**Affiliations:** 1 Department of Orthopaedic Surgery, University of Wuerzburg, Koenig-Ludwig-Haus, Brettreichstr 11, 97074 Wuerzburg, Germany; t-heinz.klh@uni-wuerzburg.de (T.H.); hrs.vasilev@gmail.com (H.V.); p-anderson.klh@uni-wuerzburg.de (P.M.A.); i-stratos.klh@uni-wuerzburg.de (I.S.); a-jakuscheit.klh@uni-wuerzburg.de (A.J.); k-horas.klh@uni-wuerzburg.de (K.H.); m-rudert.klh@uni-wuerzburg.de (M.R.); 2 Department of Orthopedics and Trauma Surgery, Musculoskeletal University Center Munich (MUM), University Hospital, LMU Munich, Marchioninistr 15, 81377 Munich, Germany; boris.holzapfel@med.uni-muenchen.de

**Keywords:** direct anterior approach (DAA), coxa profunda, protrusio acetabuli, primary total hip arthroplasty (THA)

## Abstract

Introduction: The direct anterior approach (DAA) represents a well-recognized soft tissue sparing technique for primary total hip arthroplasty (THA). The feasibility and suitability of the DAA in cases of complex acetabular deformities, namely coxa profunda (CP) and protrusio acetabuli (PA), remain to be determined. Methods: A total of 188 cases of CP (100 cases) and PA (88 cases) hips undergoing primary THA via the DAA were retrospectively analyzed. Surgical and radiographic parameters were evaluated and potential complications were assessed. Finally, successful implantation was defined if surgical and radiographic parameters were well within established values of non-complex primary THA. Results: In 159 hips, the medial border of the acetabular component was transferred laterally to the ilioischial line, corresponding to a fully treated acetabular protrusion. In 23 (12.23%) cases, mild, and in 5 (2.66%) cases, moderate residual acetabular protrusion remained after THA. Postoperatively, 11.40% (PA group) and 9.00% (CP group) had a leg length discrepancy (LLD) greater than 10 mm. The mean operative time was significantly less than 60 min. A linear relationship between the BMI and operative time was observed, with an additional 0.9 min of operative time per BMI unit. Overall, complications were rare and did not differ between the two groups. Conclusion: The results of this study suggest that the DAA is a suitable approach for primary THA in patients with coxa profunda and acetabular protrusion if performed by experienced surgeons familiar with the DAA. Obese patients with acetabular protrusion may pose a significant limitation to the DAA and caution should be advised in cases of obesity.

## 1. Introduction

Primary total hip arthroplasty (THA) has evolved to the surgical procedure of choice for the end-stage osteoarthritic hip, allowing for a high functional recovery with satisfying results [1,2,3]. Since the introduction of the modern THA by Sir John Charnley in the early 1960s, the implantation techniques, implant designs and materials have undergone consistent change in an attempt of optimizing postoperative results, patient satisfaction rates and longevity of the implants [4]. During this episode of ongoing refinement, surgical approaches to the hip joint for THA have also seen significant changes, and a variety of different exposures have been proposed, ranging from the posterior approach and the lateral approach to the anterolateral and direct anterior approach (DAA). Recently, the DAA has experienced a sharp increase through its minimally invasive and tissue sparing nature [5]. Additionally, the DAA is not a new surgical procedure, as it has already been described as early as 1881 by Carl Hueter as an approach to the hip joint [6]. In the following years, this surgical exposure has been refined by Smith-Peterson in 1917 and ultimately, in 1950, Judet and Judet first described the use of the DAA for THA [7,8]. Several key factors set the DAA apart from other existing surgical incision techniques to the hip: Firstly, it is the only approach to the hip joint that uses a real internervous und intermuscular interval by going through the natural gap between the tensor fasciae latae (TFL) and the sartorius muscle. Secondly, the supine position of the patient enables an uncomplicated use of intraoperative fluoroscopy for component position control. These potential benefits in conjunction with a postulated accelerated recovery, less postoperative pain and a probably reduced dislocation rate have further caused a high surge and widespread use of the DAA THA [9,10]. Despite the potential merits of the DAA, some limitations and challenges need to be considered including a steep learning curve with a high risk for intraoperative complications in unexperienced hands [11]. Lately, the scope of the DAA has tremendously expanded from primary THA to more complex and revision THA [12,13,14,15,16]. For instance, the DAA has already been shown to be a safe approach for primary THA in patients with anatomical abnormalities that can be encountered in cerebral palsy or following femoral or pelvic osteotomies [17]. However, proper patient selection for DAA THA remains a key factor for a successful prosthesis implantation.

It was the primary aim of this study to investigate the suitability and feasibility of the DAA for THA in patients with complex acetabular deformities, namely coxa profunda (CP) and protrusio acetabuli (PA), by analyzing radiographic and surgical data. 

## 2. Materials and Methods

### 2.1. Study Population

From September 2017 to February 2020, a total of 188 patients were retrospectively analyzed using medical records at a single university center for orthopedic surgery. Inclusion criteria were defined as follows: (1) radiographic appearance of severe osteoarthritis of the hip of at least Kellgren–Lawrence grade III, (2) clinical symptoms of hip osteoarthritis with persistent pain, impaired joint function and reduced walking distance, (3) surgical incision technique according to the DAA for THA and (4) radiographic presence of CP or PA. Any revision cases were not considered for this study. Furthermore, patients receiving a THA due to acute femoral neck fractures were excluded from this study. 

Two groups were formed and patients were either assigned to the CP or PA group, based on the pelvic radiographic appearance of the osteoarthritic hip joint. 

Postoperatively, a standardized rehabilitation protocol was applied, encompassing full-weightbearing mobilization with the usage of walking aids. Terminal flexion was restricted (no flexion > 90° for 12 weeks) and patients were advised to avoid common dislocation maneuvers (adduction and external rotation in extension and adduction and internal rotation in hyperflexion) for 12 weeks following surgery. The postoperative rehabilitation was not adapted or modified for this patient cohort and was applied to all patients of the orthopedic center receiving THA using the DAA, regardless of the degree of preoperative acetabular deformity. 

This retrospective study was presented to the local ethics committee. The need for approval has been waived.

### 2.2. Surgical Procedure

The commonly reported surgical technique of the DAA has been applied [18,19]. In detail, patients were placed in supine position and surgery was performed on a standard operation table. Prior to incision, the greater trochanter (GT) and the anterior superior iliac spine (ASIS) were identified and marked. The proximal starting point of the incision was identified by going approximately 3 cm or two finger breadths distal and lateral from the ASIS. Incision was then carried out for about 5–6 cm distally, pointing towards the lateral distal femoral condyle or fibular head. After sharp dissection of the subcutaneous tissue, the fascia of the tensor fasciae latae (TFL) was identified. The fascia was dissected in line with the fibers and the interval (Hueter interval) between the sartorius muscle and TFL was exposed. At this point, electrocauterization of the branches of lateral circumflex femoral vessels was performed. A double osteotomy of the femoral neck was than performed for removal of the femoral head, which gives access to the acetabular fossa for reaming. In cases of deep medial head protrusion, a more distal femoral neck osteotomy was performed in order to gain access for the removal of the femoral head. Abductor tenotomy could be avoided by deep muscular relaxation induced by the anesthesia shortly before head extraction was performed. Autologous cancellous bone harvested from the resected femoral head was used for augmentation in the case of a deep and insufficient medial wall. In cases of limited cup stability and severe osteoporosis, cementation of the cup was performed. Additional screw fixation was possible with the standard cup implant, but not necessary in any of the cases. After placing the acetabular cup with the insert, the proximal femur was exposed by placing a bone hook into the femoral canal and simultaneously bringing the limb into hyperextension, adduction and external rotation. The posterior capsule was released and a Mueller retractor was placed under the GT for better visualization of the femoral canal. Subsequently, broaching of the femoral canal followed until the preoperatively planned size of the femoral prothesis was reached, ensuring rotational stability. The hip joint was then reduced with the trial femoral prothesis in place. If adequate joint stability and acceptable leg length discrepancy (LLD) were achieved, the trial implant was removed and the original implant of the same size was implanted, followed by intraoperative fluoroscopic control. Before wound closure, 2 g of tranexamic acid was injected into the hip joint. 

All procedures were performed by seven senior physicians (J.A., B.H., S.B., M.R., R.S., M.W. and M.L.) and identical sets of surgical tools were used. The ML-Taper femur prothesis and the Allofit S Alloclassic acetabular cup from Zimmer Biomet were used throughout. 

### 2.3. Measurements

Radiographs of the pelvis were obtained according to a standardized protocol and checked for inclinational or rotational errors prior to templating and measuring. Radiographs were stored digitally using the PACS (Picture Archiving and Communication System) and measurements were assessed using the angle and measurement tools of the X-ray viewer (DeepUnity Review, DH Healthcare GmbH, Bonn, Germany). All radiographic measurements were performed on a plain anteroposterior pelvic X-ray. 

**Figure 1:**
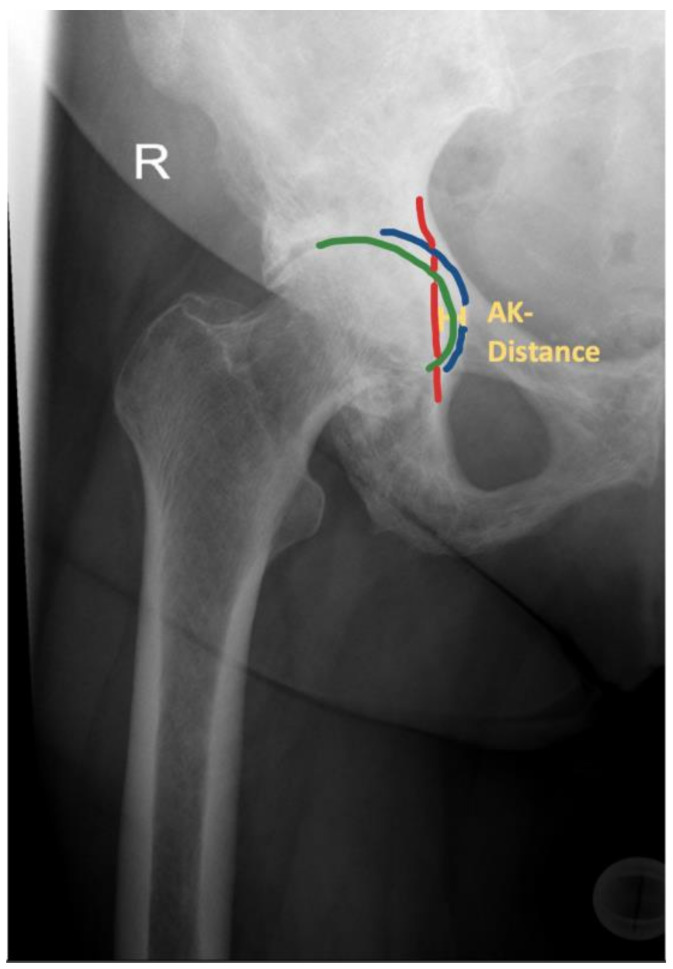
Example of a patient with acetabular protrusion of the right (R) hip. The medial cortex of the femoral head (green line) can be outlined medial to the ilioischial line (red line). The medial border of the acetabular fossa is marked in blue. The AK distance is displayed in yellow.

**Figure 2:**
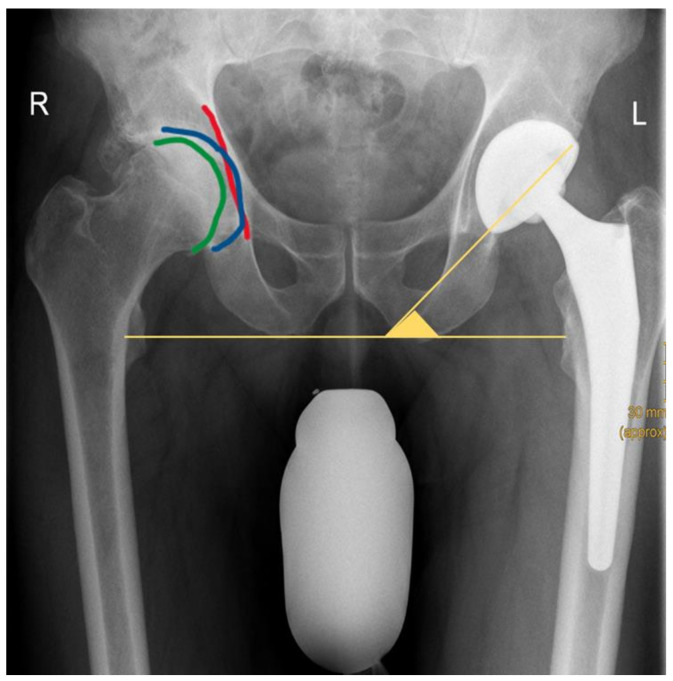
Example of a patient with coxa profunda of the right hip. The medial wall of the acetabular fossa (blue line) can be outlined medial to the ilioischial line (red line). The medial cortex of the femoral head (green line) is clearly visible lateral to the ilioischial line. Postoperatively, the cup inclination angle was measured as the angle subtended by a line tangential to the ischial tuberosities and a second line tangential to the cup rim (yellow line). R: right side, L: left side.

Differentiation between CP and PA was made based on radio-morphologic characteristics: (1) CP was assumed if the medial wall of the acetabular fossa was located medial to the ilioischial line with the medial cortex of the femoral head still lateral or in line with the ilioischial line, and (2) PA was acknowledged if the medial wall of the acetabular fossa and the medial cortex of the femoral head were both medial to the ilioischial line (Figure 1 and Figure 2) [20]. 

**Figure 3:**
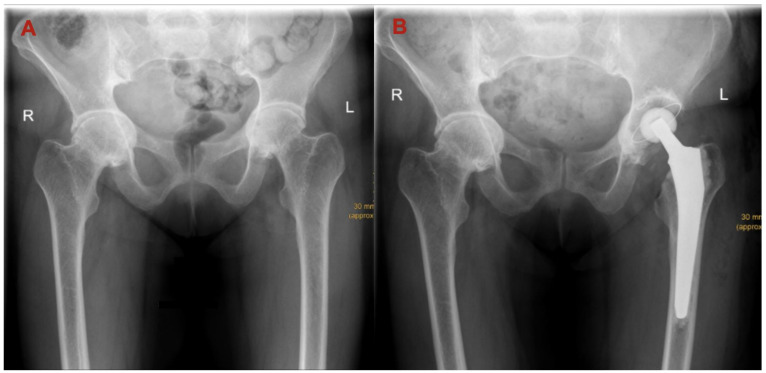
Example case of a patient with severe PA of both hips. (**A**): Preoperative finding. (**B**): Postoperative finding after THA. The medial wall of the acetabular component is slightly placed too medially despite augmentation of the medial acetabular wall with autologous cancellous bone. R: right side, L: left side.

Moreover, the severity of PA was further specified by measuring the horizontal distance between the ilioischial line (also known as Kohler line) and the medial acetabular border, denoted as AK distance (Figure 1). Postoperatively, the medial edge of the acetabular component was used as a surrogate of the medial acetabular wall (Figure 3). 

The increments were formed according to the AK distance: (1) 1 to 5 mm corresponding to mild PA, (2) 6 to 15 mm corresponding to moderate PA and (3) AK distance > 16 mm corresponding to severe PA [21].

**Figure 4:**
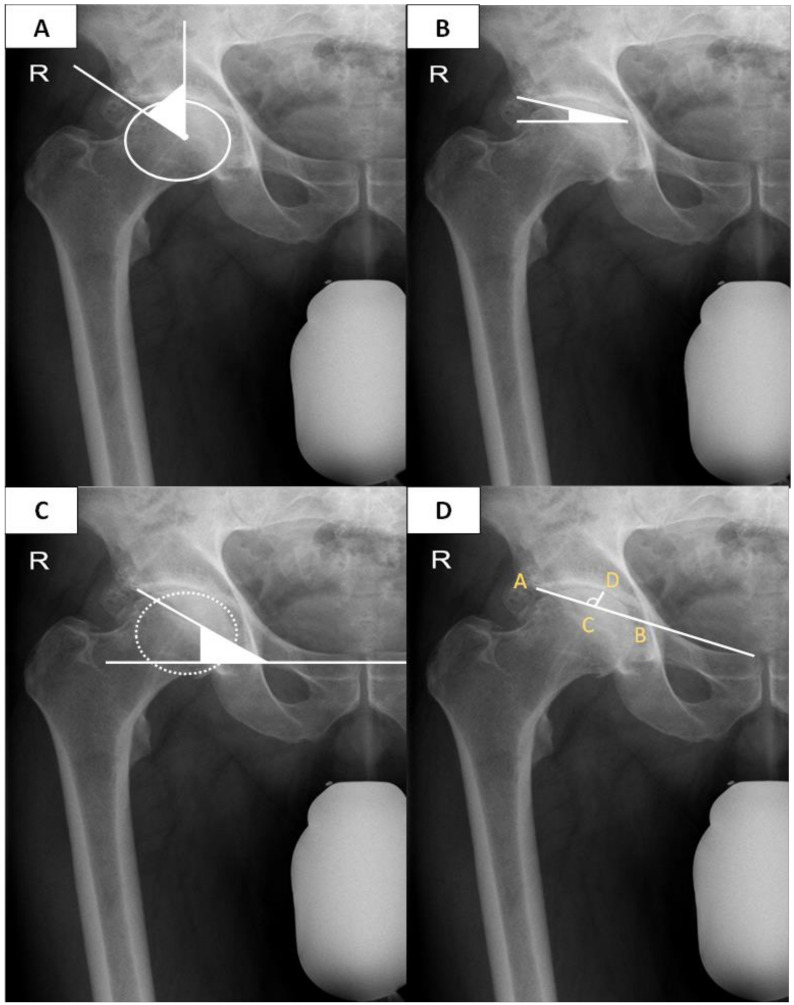
Exemplarily radiographic measurements. (**A**): Exemplarily measurement of the lateral center edge angle (LCEA). (**B**): Measurement of the Tönnis angle. (**C**): Measurement of the Sharp angle. (**D**): Measurement of the acetabular width (distance CD/AB × 1000). R: right side.

The lateral center edge angle (LCEA) was defined as the angle formed between a line from the center of the femoral head pointing towards the lateral edge of the acetabulum and a second line extending parallel to the pelvic longitudinal axis from the femoral head center [22,23]. Furthermore, the Tönnis and Sharp angles were evaluated according to established measurement guidelines [22,24]. Acetabular width denotes the line extending from the lateral sourcil to the inferior acetabular rim. Acetabular depth was measured as the perpendicular distance to the deepest point of the fossa from a line extending from the lateral sourcil towards the superior pubic angle (acetabular width line) (Figure 4). 

**Figure 5:**
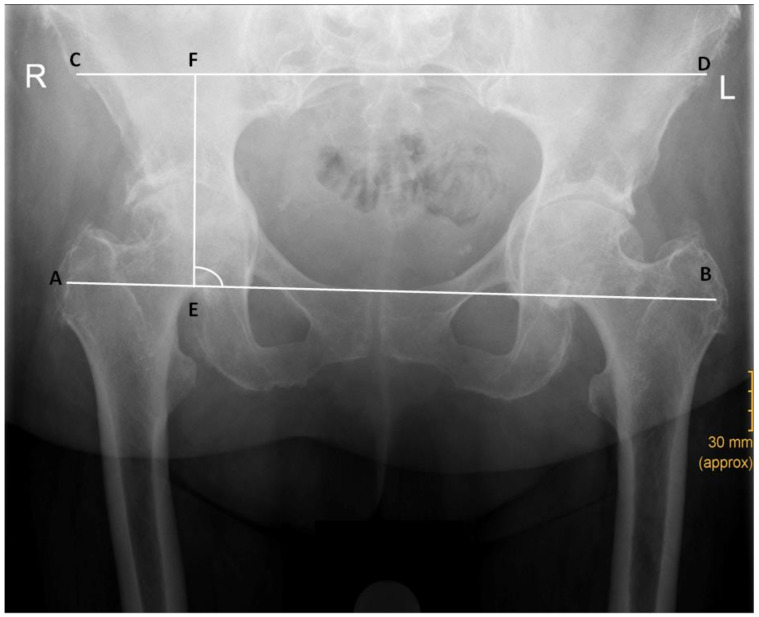
Measurement of vertical and horizontal working space. The vertical distance from the greater trochanter to the ipsilateral ASIS was defined as the vertical working space (distance EF). The ratio of the horizontal distances between both ASIS and GT was defined as the horizontal working space (distance AB/distance CD).

The acetabular depth to width ratio (ADWR) was calculated by dividing the acetabular depth by the acetabular width and multiplying the value by 1000 (ADWR = acetabular depth/acetabular width × 1000) [25,26]. Additionally, the vertical and horizontal working space for prothesis implantation was calculated. The vertical working space (AGVD) was defined as the vertical distance from the GT to the ASIS [27] (Figure 5). 

The ratio of the horizontal distance between both ASIS and both GT denoted the horizontal working space (GT/ASIS), with a smaller ratio indicating a decreased horizontal working space [27]. The horizontal distance from the center of the femoral head to the longitudinal shaft axis was defined as the femoral offset (FO) [28]. Leg-length discrepancy (LLD) was evaluated both pre- and postoperatively by measuring the difference in vertical distance from the midpoint of the lesser trochanter to the floor of the teardrop for each side. Postoperatively, cup inclination was measured as the angle between the lines tangential to the cup rim and to the ischial tuberosities (Figure 2). 

### 2.4. Statistics

Statistic calculations were performed using SPSS statistical software (SPSS, Chicago, IL, USA, Version 27). Ordinal variables were expressed as mean values and standard deviations. For categorial variables, absolute and relative frequencies were calculated. Data were checked for normal distribution using the Kolmogorov–Smirnov test. Differences between the CP and PA groups were assessed using the independent T-test or the Mann–Whitney U Test. Frequency differences of categorial variables were compared using the chi-square test. Differences within a group between different time intervals (preoperative to postoperative) were examined using the dependent T-test or Wilcoxon test. Furthermore, logistic and linear regression analysis was used to test several independent factors for their potential influence on the outcome parameters. Correlation associations of the evaluated parameters were assessed using the Pearson and Spearman correlation tests. A *p*-value of 0.05 was set as level of significance. 

## 3. Results

### 3.1. Patient Demographics

In total, 88 patients were assigned to the PA group and another 100 patients were assigned to the CP group. Both for the PA and the CP groups, a strong predominance of female patients was evident. Patient demographics and characteristics are depicted in Table 1. 

Of note, patients in the PA group showed a significantly higher age (71.52 ± 12.46 years vs. 66.96 ± 10.15 years) and Kellgren–Lawrence score (3.92 ± 0.27 vs. 3.39 ± 0.49), as well as a statistically significant lower preoperative flexion range (83.61 ± 18.88 degrees vs. 92.22 ± 15.00 degrees). The number of cemented cups was significantly higher in the PA group (Table 1).

### 3.2. Radiographic Outcomes

The mean preoperative LCEA was significantly different between the CP and PA groups, with an average higher value in the PA cohort (Table 2). 

An LCEA ≥ 47.85° was determined as the cut-off value for being highly indicative for acetabular protrusion with a sensitivity and specificity of 76.1% and 80.0%, respectively, based on the ROC analysis. Furthermore, the Tönnis angle and Sharp angle were significantly different in the CP and PA groups (Table 2). In the PA cohort, 31 patients with mild (AK distance: 1 to 5 mm), 50 patients with moderate (AK distance: 6 to 15 mm) and 3 patients with severe (AK distance: ≥15 mm) acetabular protrusion were found. Postoperatively, in 159 hips, the medial border of the acetabular component was transferred lateral to the ilioischial line, which corresponded to a fully treated acetabular protrusion. In total, 23 (12.23%) cases remained with a mild and another 5 (2.66%) cases remained with a moderate residual acetabular protrusion after THA. The acetabular depth and the ADWR were significantly higher in the PA group (Table 2). Moreover, the mean change of the femoral offset (FO) from the pre- to the postoperative visit was significantly lower in the PA group (PA: 0.71 mm ± 8.21 mm; CP: 4.12 mm ± 7.66 mm; *p* < 0.00). In detail, the mean femoral offset did not differ significantly in the PA cohort from the pre- to postoperative visit, whereas in the CP group, a significant increase in the femoral offset was noted (Table 3). 

**Figure 6:**
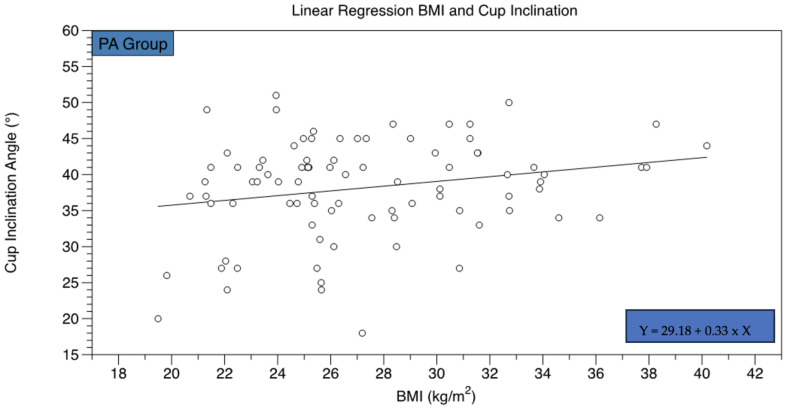
A linear association was found between the cup inclination angle and the BMI in the PA group.

Preoperative LLD was significantly higher in the PA group compared to the CP cohort (Table 2). At the postoperative visit, LLD was equal both for the CP and PA groups (Table 2). Only in cases of acetabular protrusion, a significant decrease in LLD was noted (Table 3). A LLD greater than 10 mm as measured on the postoperative radiographs was considered clinically meaningful. The number of hips with a LLD ≥ 10 mm did not differ significantly in the PA and CP groups (PA: *n* = 10 (11.40%); CP: *n* = 9 (9.00%); *p* = 0.59). The horizontal working space (GT/ASIS) was significantly decreased in the PA group compared to hips with a coxa profunda (Table 2). Likewise, the vertical working space (AGVD) was significantly lower in hips with an acetabular protrusion compared to the CP cohort (Table 2). Postoperatively, the mean cup inclination angle turned out to be significantly lower in the CP group compared to the PA cohort (PA: 38.13° ± 6.82°; CP: 36.57° ± 6.57°; *p* = 0.04). In total, 15 (PA: 17.00%; CP: 15.00%) acetabular cups were positioned outside the safe range defined as an inclination angle between 30° and 50° in the PA and CP groups, respectively. Furthermore, the linear regression analysis revealed a positive correlation regarding the BMI and the cup inclination angle only for the PA group (R^2^ = 0.23, F = 4.66, *p* = 0.03) (Figure 6).

### 3.3. Clinical Outcome 

**Figure 7:**
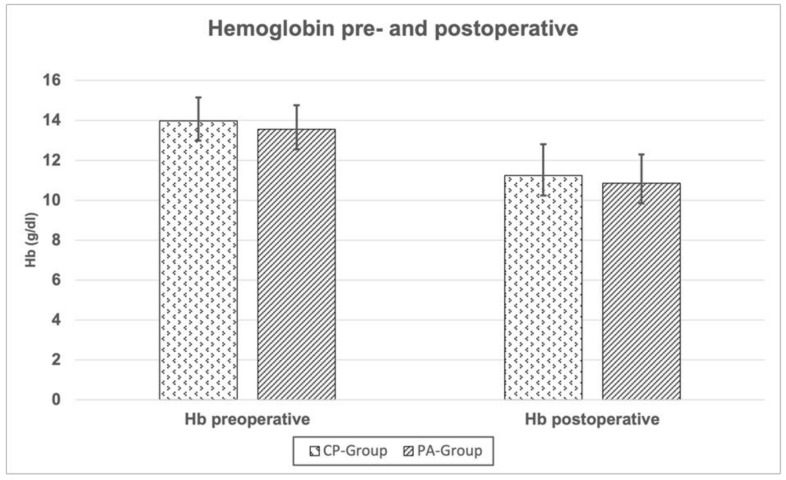
Pre- and postoperative hemoglobin values for the CP and PA groups. No statistically significant differences were found.

The difference of the preoperative (day before surgery) and postoperative (3 days after surgery) hemoglobin (Hb) was measured both for the PA and CP groups. The mean difference of the Hb did not differ between both groups (Figure 7). The mean operative time (cut to suture) did not differ between both groups (Table 4).

**Figure 8:**
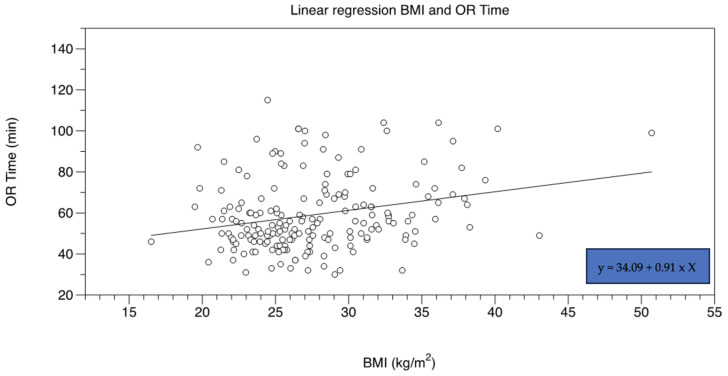
A linear association was found between the BMI and the operative time.

The mean LOS (length of stay) for both groups is depicted in Table 4. LOS did not differ for hips with CP or PA. Intraoperative and postoperative complications encompassing anemia, respiratory infections, prolonged wound healing or nerval lesions did not differ in both groups (Table 4). Two patients of the PA group were revised at 3 and 4 weeks following surgery because of acetabular cup loosening (one case) and superficial wound infection (one case). In the CP group, the femoral component had to be revised in one case because of a periprosthetic fracture occurring 4 weeks after surgery. Two patients of the CP group were readmitted after 8 weeks and one after 2 years because of periprosthetic infection. All patients were screened for readmission at the index hospital for a mean of 50.13 months. A linear association between the BMI and operation time was observed, with an additional operation time of 0.9 min per every BMI unit (R^2^ = 0.06, F(1) = 12.57, *p* = 0.01) (Figure 8).

## 4. Discussion

PA, though being a relatively rare condition in the primary osteoarthritic hip with a reported prevalence of about five percent, represents a complex hip deformity that warrants meticulous consideration prior to THA [29,30]. Several reasons render the PA a difficult and complex subject during hip replacement surgery. Firstly, the medial acetabular wall is commonly found as a thinned layer of poor bone quality, which increases the risk for generating a medial bony acetabular defect during reaming of the acetabular fossa. Secondly, through medialization of the femoral head into a deep acetabular fossa, hip motion is often significantly compromised and dislocation of the incarcerated femoral head out of the deep fossa is frequently associated with extensive soft tissue release. Thirdly, PA is many times related to a distorted anatomy, which hampers exposure and orientation during surgery. Once dislocation of the femoral head and careful preparation of the acetabular bony bed without adding any further bone deficiency have been accomplished, correct implant positioning in a way to reconstruct normal hip anatomy yields the second hitch [21,31,32]

Recently, the literature discussing PA in patients undergoing THA has mainly focused on different implantation techniques for addressing the contained medial acetabular defect (a solid graft and morselized autologous impacting grafting) and on different implant materials (porus-coated cementless cups, threaded cups and cemented cups). However, the suitability and feasibility of the aspiring minimally invasive DAA have not yet been investigated in patients undergoing THA for end-stage PA osteoarthritis. So far, the feasibility of DAA has already been linked successfully to revision THA [14,16,33]. Transferring the potential benefits of the DAA to complex hip arthroplasty in patients with PA would therefore pose an interesting and promising approach. 

As a result of this study, the analysis of the postoperative radiographs showed parameters that were well in line with those reported and acknowledged for successful total hip replacement. The mean acetabular inclination angle of the study cohort was shown to be within the safe range according to Lewinnek et al. both for the PA and CP hip [34]. Furthermore, the protrusion of the medial acetabular wall was fully corrected in most of the cases (160 out of 188 hips), only leaving about 15% of the treated hips with a slightly too medial cup placement. The mean postoperative radiographic leg length difference was well below 1 cm, which is normally well tolerated without warranting further revision surgery or shoe correction [35,36,37]. Vanrusselt et al. also defined a LLD below 1 cm in the postoperative radiographs as a sign of successful treatment [38]. A THA perioperative blood loss of about 700 to 1200 mL is commonly reported, which roughly transfers to a hemoglobin drop of about 2 to 4 units (g/dL) [39,40]. The mean perioperative blood loss of this study cohort (both PA and CP groups) is therefore well in line with reported standard values of non-complex THA. An operative time > 120 min has been associated with a higher overall complication rate for THA [41]. In this study cohort, both the profunda hips and the PA hips showed a mean operative time of about 60 min, which is remarkedly shorter than standard operative times reported in the literature [42]. Moreover, the femoral offset was adequately reconstructed in most of the cases. However, the BMI turned out to be an independent factor of the mean operative time, with a mean increase in the operative time of 0.9 min per every BMI unit. This may be attributable to a relatively narrow working space and limited capability of exposure through the DAA. Sang et al. also demonstrated significantly higher operative times with higher BMI values in THA through the DAA [27]. Furthermore, study results demonstrated that higher BMI values pose to the surgeon a risk of accidentally increasing the cup inclination angle, probably due to limited visualization and an impeding soft tissue envelope in obese patients. 

Of note, due to the retrospective study design, there are inevitably some shortcomings to this study such as the lack of a control group for comparison. Furthermore, the analysis of radiographic parameters poses the risk of measuring errors and thereby over- or underestimating associations. However, to our knowledge, this is the very first study investigating the feasibility and suitability of the DAA for complex THA in PA. Furthermore, the high number of patients with 100 hips investigated in the PA group adds further strength. Moreover, this is the very first study simultaneously considering CP hips in THA using the DAA. 

## 5. Conclusions

The results of this study suggest that the DAA is a suitable approach for primary THA in patients with coxa profunda and acetabular protrusion if performed by experienced surgeons familiar with the DAA. Obese patients with acetabular protrusion may pose a significant limitation to the DAA and caution should be advised in cases of obesity.

## Figures and Tables

**Table 1:** Patient demographics and characteristics of the CP and PA groups.

		Mean (±SD) or Relative Frequency (%)	*p*-Value (CP Group vs. PA Group)
PA Group	Sex (m/f)	14.80%/85.20%	0.55
	Age, years (a)	71.52 + 12.46	0.01
	BMI (kg/m^2^)	27.24 ± 4.61	0.36
	VAS, preoperative	5.93 ± 2.14	0.95
	Hip flexion, preoperative (degrees)	83.61° ± 18.88°	0.00
	Hip abduction, preoperative (degrees)	13.03° ± 8.33°	0.00
	Hip adduction, preoperative (degrees)	10.43° ± 7.35°	0.53
	Hip flexion, postoperative (degrees)	84.70° ± 8.20°	0.06
	Cemented cups	5 (5.68%)	0.02
	Sex (m/f)	18.0%/82.0%	0.55
CP Group	Age, years (a)	66.96 ± 10.15	0.01
	BMI (kg/m^2^)	27.91 ± 5.25	0.36
	VAS, preoperative	5.90 ± 2.29	0.95
	Hip flexion, preoperative (degrees)	92.22 ± 15.00	0.00
	Hip abduction, preoperative (degrees)	18.72° ± 10.95°	0.00
	Hip adduction, preoperative (degrees)	11.38° ± 8.21°	0.53
	Hip flexion, postoperative (degrees)	86.76° ± 5.76°	0.06
	Cemented cups	0 (0.00%)	0.02

**Table 2:** Radiographic parameters of the CP and PA groups. The *p*-value was calculated for statistical difference between the groups.

Radiographic Parameter	CP Group	PA Group	*p*-Value (CP vs. PA)
LCEA (mean ± SD)	40.41° ± 9.82°	55.09° ± 9.64°	<0.00
Sharp (mean ± SD)	36.44° ± 5.15°	34.04° ± 4.81°	<0.00
Tönnis (mean ± SD)	6.16° ± 5.70°	3.12° ± 5.89°	<0.00
Acetabular depth (mean ± SD)	24.73 mm ± 4.01 mm	27.92 mm ± 4.67 mm	<0.00
ADWR (mean ± SD)	334.60 ± 43.33	388.50 ± 61.22	<0.00
Change of FO (preoperative vs. postoperative) (mean ± SD)	4.11 mm ± 7.66 mm	0.71 mm ± 8.21 mm	<0.00
FO, preoperative (mean ± SD)	42.94 mm ± 7.20 mm	46.22 mm ± 7.57 mm	<0.00
FO, postoperative	47.05 mm ± 5.38 mm	45.52 mm ± 4.46 mm	0.03
LLD, preoperative (mean ± SD)	4.49 mm ± 4.24 mm	7.65 mm ± 6.76 mm	<0.00
LLD, postoperative (mean ± SD)	4.87 mm ± 4.12 mm	5.41 mm ± 5.00 mm	0.41
AGVD (mean ± SD)	108.59 mm ± 13.75 mm	101.91 mm ± 14.36 mm	<0.00
GT/ASIS (mean ± SD)	1.19 ± 0.07	1.13 ± 0.07	<0.00
Cup inclination	36.57° ± 6.57°	38.13° ± 6.82°	0.04

**Table 3:** Change of the femoral offset (FO) and leg-length discrepancy (LLD) within the CP and PA groups from the preoperative to the postoperative visit.

		Preoperative Value	Postoperative Value	*p*-Value
CP Group	FO (mean ± SD)	42.94 mm ± 7.20 mm	47.05 mm ± 5.38 mm	<0.00
	LLD (mean ± SD)	4.49 mm ± 4.24 mm	4.87 mm + 4.12 mm	0.48
PA Group	FO (mean ± SD)	46.22 mm ± 7.57 mm	45.52 mm ± 4.46 mm	0.42
	LLD (mean ± SD)	7.65 mm ± 6.76 mm	5.41 mm ± 5.00 mm	0.02

**Table 4:** Surgical outcome values of the CP and PA groups. *p*-values were calculated for differences between both groups.

	CP Group	PA Group	*p*-Value
Change of Hb (preoperative vs. postoperative) (mean ± SD)	2.73 g/dL ± 1.22 g/dL	2.66 g/dL ± 1.18 g/dL	0.71
Operative time (mean ± SD)	59.62 min ± 17.78 min	58.50 min ± 17.81 min	0.67
LOS (days) (mean ± SD)	8.44 ± 2.09	9.11 ± 3.58	0.11
Complication rates (total number *n*, percent)			
Postoperative anemia	3 (3.00%)	2 (2.27%)	0.54
Prolonged wound healing	4 (4.00%)	4 (4.54%)	
Postoperative regional paresthesia	2 (2.00%)	1 (1.14%)	
Respiratory infection Intraoperative fracture (femur or acetabulum)	2 (2.00%) 1 (0.53%)	2 (2.27%) 2 (1.06%)	

## Data Availability

Data can be obtained from the authors upon reasonable request.
